# Change in self-reported somatic symptoms among patients in opioid maintenance treatment from baseline to 1-year follow-up

**DOI:** 10.1186/s12888-024-05590-w

**Published:** 2024-02-21

**Authors:** Endre Dahlen Bjørnestad, John-Kåre Vederhus, Thomas Clausen

**Affiliations:** 1 Addiction Unit, Sørlandet Hospital HF, Po. box 416, Kristiansand, Norway; 2 Norwegian Centre for Addiction Research (SERAF), University of Oslo, Kirkeveien 166, N-0407 Oslo, Norway

**Keywords:** Opioid maintenance treatment, Somatic symptoms, Somatic conditions

## Abstract

**Background:**

High somatic comorbidity is common among patients in treatment for opioid use disorder (OUD). The present study aims to investigate changes in self-reported somatic health conditions and somatic symptoms among patients entering opioid maintenance treatment (OMT) programs.

**Methods:**

We used data from the Norwegian Cohort of Patients in OMT and Other Drug Treatment (NorComt) study. Of 283 patients who entered OMT, 176 were included for analysis at a 1-year follow-up. Participants provided self-reported data during structured interviews on somatic conditions, somatic symptoms, substance use severity measures, and mental distress. A multivariable linear regression analysis identified factors associated with changes in the burden of somatic symptoms.

**Results:**

Patients entering OMT reported a high prevalence of somatic conditions at the beginning of treatment, with 3 of 5 patients reporting at least one. The most prevalent condition was hepatitis C, followed by asthma and high blood pressure. Patients reported experiencing a high number of somatic symptoms. The intensity of these symptoms varied across a wide spectrum, with oral health complaints and reduced memory perceived as the most problematic. Overall, for the entire sample, there was no significant change in somatic symptoms from baseline to 1 year. Further analysis indicated that those who reported a higher burden of somatic symptoms at baseline had the greatest improvement at the 1-year follow-up. A higher number of somatic conditions and higher mental distress at baseline was associated with improvements in somatic symptoms burden at follow-up.

**Conclusions:**

Patients in OMT report a range of somatic conditions and somatic symptoms. Given the wide range of symptoms reported by patients in OMT, including some at high intensity levels, healthcare providers should take into consideration the somatic healthcare needs of individuals in OMT populations.

**Clinical trial registration:**

Clinicaltrials.gov no. NCT05182918. Registered 10/01/2022 (the study was retrospectively registered).

**Supplementary Information:**

The online version contains supplementary material available at 10.1186/s12888-024-05590-w.

## Background

Opioid maintenance treatment (OMT) is an evidence-based treatment aimed at reducing harmful consequences associated with non-medical opioid use [1]. OMT reduces the risk of fatal overdose and is also partly protective of other causes of death [2–5]. High somatic comorbidity is common among patients in OMT treatment, and patients in OMT are at increased risk of developing comorbidities, such as respiratory and cardiovascular diseases (CVDs) [6–9]. High levels of smoking and smoking-related harms are particularly prominent in OMT populations [8, 10, 11]. Thus, somatic disease is a major concern for these patients, and non-drug-related premature causes of death are more prevalent than in the general population [5, 12–15]. Individuals with opioid use disorder (OUD) are often affected by a wide range of somatic ailments but are sometimes less likely to seek regular health care check-ups [16, 17]. Furthermore, individuals with OUD are over-represented in terms of acute health care utilization compared to the general population but may still be underserved in terms of their long-term somatic needs [17, 18]. Patients also face barriers to seeking health care, such as stigma, which may negatively affect somatic health outcomes [19]. Patients in OMT report lower health-related quality of life [20], and somatic symptoms, such as pain, have been associated with medical and psychological comorbidities [21, 22]. A previous publication on a subsample of older patients in OMT from the Norwegian Cohort of Patients in OMT and Other Drug Treatment (NorComt) study reported an association between mental distress and chronic conditions and levels of somatic symptoms [10]. Individuals with OUD in OMT are more likely to reach a higher age than individuals with OUD who are not in OMT [3, 5, 23]. After implementing OMT, many countries experience aging OMT populations [23, 24]. Healthcare providers are likely to be challenged in providing treatment services for aging OMT patients with complex needs [25, 26]. To further improve the treatment services for patients, we need more knowledge about their somatic status and the needs of patients as they age while in treatment.

The objective of this study was to examine the changes in somatic health among patients enrolled in OMT programs over a span of about 1 year. We assessed self-reported somatic conditions and the perceived burden of somatic symptoms at the beginning of treatment (T0) and again after 12 months (T1) follow-up. Additionally, we explored factors associated with improvement in the burden of somatic symptoms at T1.

## Methods

### Study design

This study used data from the NorComt study, a longitudinal, naturalistic, multi-site study [27–29].

### Setting

Participants were recruited from participating OMT facilities across Norway during 2012–2015. Follow-up data were collected during 2014–2016. In Norway, OMT is mostly provided on an outpatient basis by publicly funded health services and follows national treatment guidelines [30]. An established OUD diagnosis is the main criteria for entering treatment. The specialist healthcare service serves as the overall responsible provider, but treatment is provided in collaboration with primary healthcare and social services. The OMT guidelines in use during the data collection period were implemented in 2010. They have since been revised, in May 2022 [30]. More details about the study setting were provided in previous publications [27–29].

### Participants

Initially, 283 patients who entered OMT participated in the study. The formal inclusion criterion was admittance to an OMT facility. There were no formal exclusion criteria. Participants were consecutively enrolled in the study when beginning treatment and consented at baseline (T0) to be contacted for additional data collection 1 year later (T1) for a follow-up interview [29]. Clinicians at each treatment center conducted the baseline interviews with enrolled patients within 3 weeks from treatment initiation. In preparation for the interviews the NorComt research group provided training to facility staff through in-person sessions, and provided support throughout the T0 data collection phase. Follow-up data were collected approximately 12 months (range 11–18 months) following inclusion. At T1, the interviews were conducted by three doctoral students and two trained and experienced interviewers from the NorComt research group. Of the 283 patients who entered OMT, 176 (62%) were included in the analysis and 107 (38%) were coded as lost to follow-up (see flow chart describing reasons for being lost to follow-up in Supplementary file 1).

### Measures

The study used a structured interview that included questions on sociodemographic variables, housing, substance use, and a variety of measurements relevant to the treatment of substance use disorders (SUDs) [27–29]. To assess the severity of somatic symptoms, a checklist consisting of 16 common somatic symptoms among individuals with SUD was utilized [16]. The checklist drew inspiration from validated scales such as the Patient Health Questionnaire 15-Item Somatic Symptom Severity Scale (PHQ-15), and other validated scales [31, 32]. The items were selected based on their relevance to symptoms that, according to clinical judgment, were deemed most relevant for patients in OMT. These were: oral health complaints (teeth/gum), reduced memory, indigestion, constipation, joint pains, headaches, dizziness, respiratory ailments, visual disturbances, urine tract ailments, chest pains, skin infections, diarrhea, blood clots, eczema, sexually transmitted diseases (STDs). Participants were asked to indicate the extent to which they had been bothered in the past 6 months by each symptom. Answers were presented on a 0–4 response format, with 0 corresponding to “not at all”, 1 “a little”, 2 “moderately”, 3 “a lot”, and 4 “very much”. Patients were also asked to indicate whether the symptoms were chronic (i.e., had lasted at least 3 months in the past 6 months) using “yes” or “no”. The main outcome was burden of somatic symptoms. This was calculated as the sum of numeric responses on the 16 somatic symptoms variables, resulting in a range from 0 to 64.

Patients were asked to indicate whether they currently had one or more common somatic diseases/conditions on a list of 10: diabetes, high blood pressure, CVD, chronic obstructive pulmonary disease (COPD), asthma, hepatitis B, hepatitis C, liver cirrhosis, human immunodeficiency virus (HIV), and cancer. If they replied “yes”, they were asked whether they had received treatment for the given condition in the past 6 months. To assess the severity of substance use, we utilized several substance-related variables, including the Severity of Dependence Scale (SDS), intravenous use in the past 6 months, age at first intravenous use, number of substances used in the past 6 months, and smoking (tobacco) in the past 6 months [8, 15, 33, 34]. The SDS is a validated five-item scale that was originally designed to measure dependence on specific substances but was rephrased to reflect the overall dependence on substances (e.g., “Did you think your use of substances was out of control?”). Responses were given in a 4-point format ranging from 0 to 3, with 0 corresponding to “Never” and 3 corresponding to “Always”. The summed scale ranged from 0 to 15, with higher scores representing higher severity. In addition to the SDS, injection drug use was incorporated as a proxy for assessing the severity of substance use in the regression analysis [34, 35]. Mental distress was assessed with the Hopkins Symptom Checklist 25 (HSCL-25) which included common symptoms of mental distress experienced in the past week. The scale had a 5-point Likert response format with higher scores representing higher severity [36, 37].

### Statistical analysis/analysis strategy

To investigate whether there were differences in the somatic symptom burden at T0 based on age, we divided the included participants into three groups stratified by age: <40, 40 to 50, and ≥ 50. For descriptive analysis, we used a one-way between-groups analysis of variance (ANOVA). Change in burden of somatic symptoms was tested using paired t-tests. Post-hoc correction for multiple testing was not utilized [38]. To further explore changes, we divided the sample into three equal-sized groups and conducted an exploratory descriptive analysis on change based on low, moderate, and high somatic symptom scores at baseline. To investigate factors associated with improvements in the burden of somatic symptoms from T0 to T1, a multiple linear regression with simultaneous entry of variables (the ‘‘enter’’ method) was used. Preliminary analysis were conducted to assess that assumptions for the analysis were met, such as normality, linearity and multicollinearity. We used the change in the somatic symptoms score as the dependent variable. To calculate this, we subtracted the somatic symptoms score at T1 from the somatic symptoms score at T0, resulting in a differential sum score Thus, a negative number represented worsening, zero represented no change, and a positive number represented improvement. Socio-demographic and clinical severity variables at T0 were included as independent variables. Results are presented as unstandardized beta coefficients with 95% confidence intervals (CIs). The R-squared (R^2^) value assessed the proportion of variability in the dataset. Analyses of variables were considered to be significant at a p value < 0.05. All analyses were performed using IBM SPSS Statistics version 28.

## Results

Baseline characteristics for the full sample (*N* = 283) at T0, grouped by included (*N* = 176) and lost to follow-up (*N* = 107) at T1, are presented in Table 1. For both the included and lost to follow-up-groups, the mean age was 39 ± 10, and roughly 70% were male. Baseline characteristics were similar between those who were included and those who were lost to follow-up. The examination of attrition did not uncover any significant distinctions between the two groups concerning baseline characteristics and the main outcome; burden of somatic symptoms (Table 1), nor in the proportions of somatic conditions (Supplementary file 2). At T1, 92% (*N* = 162) of the included participants were still in treatment.

**Table 1:** Baseline characteristics, demographics, substance use, and other relevant variables for patients entering OMT (*N* = 283)

	Included (*N* = 176)	Lost to follow-up^f^ (*N* = 107)	*p*-value^a^
**Characteristics**			
Age, years	39 ± 10	39 ± 10	0.953
Male	127 (72)	75 (70)	0.813
Completed lower secondary education	83 (47)	41 (38)	0.170
Employed or enrolled in education	25 (15)	7 (7)	0.073
**Treatment variables**			
Buprenorphine^b^	43 (24)	28 (26)	0.853
Buprenorphine and Naloxone^c^	99 (56)	55 (51)	0.502
Methadone	35 (20)	21 (20)	1.000
Other/unknown	0	3	-
**Substance use related variables**			
Severity of dependence^d^	10.35 ± 3.19	10.48 ± 2.83	0.735
Intravenous use past 6 months	130 (74)	78 (73)	0.968
Age at first intravenous use, years	19 ± 9	20 ± 8	0.873
No. substances past 6 months	3.8 ± 2.6	4.2 ± 3.3	0.268
Smoking (tobacco) past 6 months	166 (95)	93 (93)	0.805
**Physical health**			
Number of somatic conditions	1 (1.1)	1.2 (1.1)	0.125
Burden of somatic symptoms	13.4 (9)	14.4 (9)	0.372
**Mental health**			
Mental distress^e^	1.25 ± 0.87	1.30 ± 0.85	0.609
Attempted suicide (lifetime)	66 (38)	47 (45)	0.282

Values are given as mean ± SD or n (%). ^a^Based on t-tests and chi-squared, Yates’ correction for 2 × 2. ^b^Subutex; ^c^Suboxone; ^d^SDS; ^e^HSCL-25. ^f^Also includes cases that did not have any data on somatic symptoms.

Missing data, included group: Substances past 6 months, *N* = 3; Age intravenous, *N* = 4; Completed lower secondary education, *N* = 1; *N* = 4; Employed/edu, *N* = 4; Smoking, *N* = 1. Missing data lost to follow-up group: Employed/edu, *N* = 2; SDS, *N* = 1; Age intravenous, *N* = 1; Smoking, *N* = 1; Somatic symptoms burden, *N* = 1; Attempted suicide, *N* = 2.

### Somatic conditions

Among the 176 patients, 110 (63%) reported at least one somatic condition at T0. Self-reported somatic conditions for both T0 and T1 are presented in Table 2.

**Table 2:** Self-reported somatic conditions (*N* = 176)

	T0		T1	
Condition type:	n (%)	Treatment for condition past 6 months^a^	n (%)	Treatment for condition past 6 months^a^
Hepatitis C	82 (47)	1 (1)	84 (48)	10 (12)
Asthma	32 (18)	15 (47)	35 (20)	22 (63)
High blood pressure	15 (9)	9 (60)	20 (11)	12 (60)
CVD	13 (7)	7 (54)	11 (6)	7 (64)
Hepatitis B (*N* = 175)	12 (7)	0 (0)	11 (6)	2 (18)
COPD	11 (6)	3 (27)	11 (6)	5 (45)
Diabetes (*N* = 175)	5 (3)	4 (80)	6 (3)	3 (50)
Liver cirrhosis (*N* = 174)	4 (2)	2 (50)	3 (2)	1 (33)
HIV	3 (2)	2 (67)	5 (3)	1 (20)
Cancer	1 (1)	1 (100)	2 (1)	0 (0)

CVD, cardiovascular disease; COPD, chronic obstructive pulmonary disease; HIV, human immunodeficiency virus; ^a^ % of those that reported condition

The most frequently reported somatic conditions at T0 were hepatitis C (47%), followed by asthma (18%) and high blood pressure (9%). COPD was reported by approximately 6%, and around 1 in 3 patients reported elevated levels of respiratory ailments, suggesting elevated levels of lung disease. Around 1 in 10 of patients reported CVD. The proportions of self-reported somatic conditions remained similar at T0 and T1. Whether patients reported having received treatment for their condition varied, and for some conditions a very low proportion of patients reported treatment, particularly for hepatitis C. At T0, only 1 patient reported treatment for this condition, whereas at T1, this had increased to 10 patients. For most of the other listed conditions, more than half of the patients reported having received treatment.

#### Self-reported somatic symptoms

**Fig. 1:**
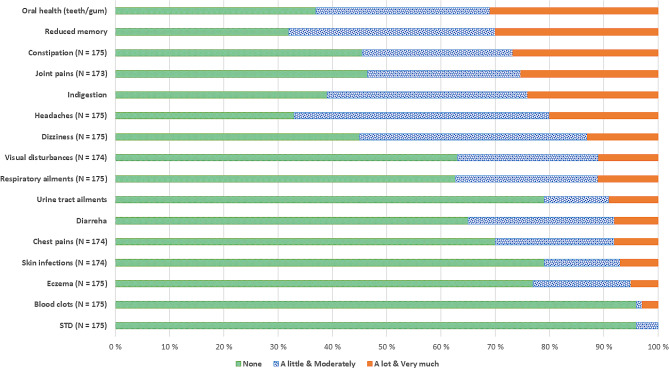
Somatic symptoms at start of treatment (T0) **Note**: Somatic symptoms. “How bothered are you by each of the following?” (NorComt study, Norway)

The prevalence of somatic symptoms at baseline is presented in Fig. 1. Patients reported a mean of 6.3 ± 3.2 and median of 6, interquartile range (IQR) 5 somatic symptoms across the organ systems/16 items at T0. Patients reported a mean 3.9 ± 4.1 (median 3, IQR 7) of the somatic symptoms as having lasted at least 3 months. Of those who reported at least being bothered “A little”, the most commonly reported symptoms were reduced memory (68%), headaches (67%), oral health complaints (63%), and indigestion (61%). The symptoms with the highest severity (i.e., patients reported being bothered a lot and very much by) were oral health complaints and reduced memory, and were reported by nearly 1 in 3 patients. Around 1 in 4 patients reported being bothered a lot or very much by joint pains, constipation, and indigestion. About 1 in 5 patients reported being bothered a lot and very much by headache (Fig. 1.). There were no significant differences on somatic symptoms burden between the age groups: F (2, 173) = 0.31, *p* = 0.732. The mean somatic symptom burdens for the age groups < 40 (*N* = 105), 40 to 50 (*N* = 42), and ≥ 50 (*N* = 29) were 13.3 (± 8.4), 14.2 (± 9.7), and 12.6 (± 10.1), respectively.

### Change in burden of somatic symptoms from T0 to T1

**Fig. 2:**
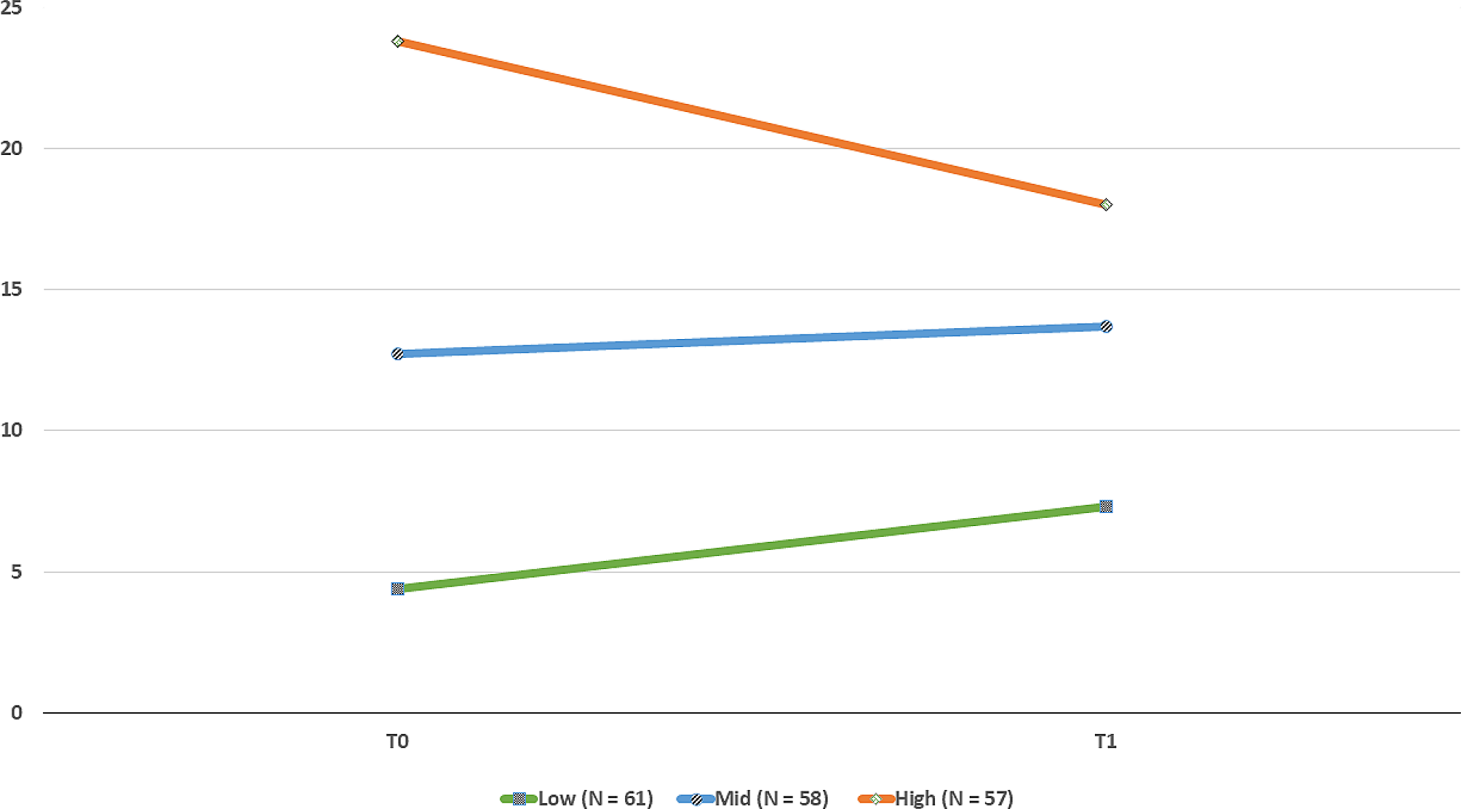
Change in somatic symptoms burden from T0 to T1 **Note**: Changes in somatic symptoms, from baseline (T0) to follow-up (T1) based on the high (*n* = 57), moderate (*n* = 58), and low (*n* = 61) segments of the somatic symptoms score at T0. *Orange line* indicates means of patients with the highest level of symptoms, *blue line* indicates means of patients with mid-scores, and *green line* indicates means of patients with low scores

There was no significant overall change in the full sample from T0 to T1, with a mean difference of 0.55 (95% CI: -0.87 to 2; *p* = 0.444). When splitting the sample into three roughly equal groups based on their score at T0 (Fig. 2), the group that reported higher somatic symptoms burden at T0 had the greatest improvement, i.e., a mean reduction of 5.8 (95% CI: 2.8 to 8.8; *p* < 0.001). However, they still reported levels above those of the other two groups at T1. The group with lower somatic symptoms burden at T0 actually experienced a slight worsening (i.e., had a higher score at T1 with a mean difference of -2.9 [95% CI: -4.6 to -1.3], *p* < 0.001). The group with scores in the mid-range did not report significant changes in somatic symptoms burden, with a mean difference of -0.9 from T0 to T1 (95% CI: -3.1 to 1.2; *p* = 0.385).

### Factors associated with improvement in the burden of somatic symptoms

An examination of factors associated with a change in burden of somatic symptoms at follow-up is shown in Table 3. In addition to the severity variables, age and sex were included as independent variables. Number of somatic conditions (β = 1.69, *p* = 0.015) and mental distress (β = 2.36, *p* = 0.007) were associated with a change in somatic symptoms burden at T1. The adjusted R^2^ for the model was 9%.

**Table 3:** Multivariable regression of covariates relevant to a change in somatic health from T0 to T1ab

Variables	Multivariable analysis	
	β (95% CI)	*p* value
Age	0.03 (-0.13/0.18)	0.747
Sex^c^	1.56 (-1.60/4.73)	0.332
Severity of dependence^d^	0.16 (-0.30/0.63)	0.488
Mental distress^e^	2.36 (0.66/4.06)	**0.007**
Injection use	1.49 (-1.81/4.78)	0.375
Number of somatic conditions	1.69 (0.33/3.06)	**0.015**

^a^Multiple linear regression with simultaneous entry of variables (the ‘‘enter’’ method); unstandardized beta coefficient with 95% confidence interval (CI).

^b^Dependent variable is difference in somatic symptoms T0-T1.

^c^Male was the reference category.

^d^SDS, Severity of Dependence Scale.

^e^HSCL-25, Hopkins Symptom Checklist-25.

## Discussion

Patients entering OMT reported a high prevalence of somatic conditions at the beginning of treatment, with 3 in 5 patients reporting at least last one. The most prevalent condition was hepatitis C, followed by asthma and high blood pressure. The prevalence of somatic conditions was similar at the 1-year follow-up. Patients reported high levels on several somatic symptoms at T0, including reduced memory, oral health, joint pains, indigestion, and constipation. Contrary to expectations, we did not find age differences on reported somatic symptoms at baseline. There was no significant change in burden of somatic symptoms from T0 to T1, but an exploratory analysis showed that those who reported a higher severity, i.e., a higher somatic symptom score at T0 had the greatest improvement. A greater number of somatic conditions and higher mental distress at T0 was associated with an improvement in burden of somatic symptoms at follow-up. Age did not contribute significantly to change in somatic symptom burden.

The proportion of patients reporting one or more somatic conditions was high at treatment entry and similar at follow-up. As a comparative example, asthma was much more prevalent in the present sample at 18% compared to findings of 5% in the Norwegian adult general population [39]. Approximately half of patients reported hepatitis C, similar to previous reports of a prevalence of 40–50% for hepatitis C in OMT populations from the same period as our data collection [40, 41]. In the present study, there was a solid increase in the percentage of patients receiving hepatitis C treatment at T1, with 12% having received such treatment in the past 6 months. A newly published report from the Norwegian Institute of Public Health indicated that Norway has come a long way in eradicating hepatitis C in the general population, and that the prevalence among people who inject drugs (PWID) decreased substantially since 2016 [42]. More patients currently receive hepatitis C treatment than in previous years [24, 43, 44]. Based on the latest Norwegian OMT status report, > 35% of patients in Norwegian OMT have completed treatment for hepatitis C, in addition to nearly 40% not having active disease based on antigen testing. There are still patients who are in need of treatment, but this suggests that it is possible, even for complex issues and hard to reach populations, to make a difference in a relatively short time given a systematic and structured approach and focused priority.

The majority of patients were bothered by one or more somatic symptoms and reported a mid to low score overall on the somatic symptoms burden index. The symptoms with the highest levels of severity were oral health complaints and reduced memory. A recent study on oral health in a similar population also reported generally poor oral health and oral health-related quality of life among OMT patients [45]. Measures have been implemented in the Norwegian context to improve oral health for patients; patients in OMT are entitled to necessary dental care from the public dental service at no cost. Nevertheless, previous research has highlighted that patients also face non-financial barriers to seeking treatment for oral ailments [46].

The issue of opioid use and the negative impact on neuropsychological functions, including memory, is well known [47]. In the present study, approximately one-third of patients had complaints about reduced memory. Whether such impairment can be attributed specifically to the OMT medication (i.e. compared to individuals with OUD outside treatment) remains unclear according to a relatively recent systematic review [48]. Polysubstance use is common among individuals with OUD, including benzodiazepine and stimulants [47], and may play a part in explaining the elevated levels of reduced memory symptoms. Cognitive impairments have previously been reported to have negative implications for treatment retention, which is of particular concern in OMT treatment [47, 49].

Many patients also reported being bothered by gastrointestinal symptoms, such as constipation and indigestion. These are common side effects associated with opioid use in general, as well as having been specifically reported in OMT-populations [50, 51]. The topic of medication side effects should be a regular discussion point during clinical encounters for the OMT population.

### Factors associated with changes in somatic symptoms

Although there was little change in the somatic symptom score for this OMT group as a whole, our regression analysis revealed that those who had the highest number of somatic conditions and higher mental distress at T0 had the largest improvement in somatic symptoms. Previous research has highlighted the link between psychological distress to pain and elevated levels of somatization [22]. Chronic disease and alleviation of mental distress are phenomena that are likely to be addressed by OMT providers, so our finding is encouraging. Though the improvements in scores were relatively modest, they may be clinically meaningful given the subjective nature of experiencing such symptoms over time and may indicate that OMT offers stabilization on the somatic side of the overall burden of OUD.

### Clinical implications

Our findings suggest that some patients are greatly bothered by a variety of somatic symptoms. In addition to agonist treatment and psychosocial treatment and support, somatic follow-up constitutes an important part of service provision for general practitioners and OMT providers treating patients in OMT. The range of symptoms and conditions that are reported by the patients are likely to affect quality of life negatively [20]. Conversely, treatment may positively affect quality of life [52]. Efforts to prevent and strengthen early diagnosis, treatment, and follow-up of chronic somatic disorders are important to reduce premature mortality and increase quality of life, thereby providing a good treatment experience [14]. In terms of oral health and gastrointestinal well-being, treatment providers should not overlook such “ordinary” symptoms and regularly follow-up on such complaints to increase the well-being of patients.

In the present study, the patient’s age was not associated with improvements in the somatic symptoms burden, indicating that the older patients were not worse off than the younger patients. However, symptoms were reported at elevated levels and indicate that the somatic health follow-up should be structurally integrated into treatment to meet patients’ somatic needs, including issues related to cognitive functioning. Clinical practice should expect that OMT patients may experience somatic symptoms and disease at younger ages than non-SUD patients [20, 53]. It is important for treatment services to address the future burden of somatic symptoms that may be exacerbated by the presence of chronic conditions in aging OMT populations [27]. The revised national OMT guideline more directly notes that specialist healthcare can play a role in the somatic follow-up of patients, whereas the old guidelines highlighted the role of primary care (i.e., general practitioners) [54]. The high relative prevalence of somatic conditions and symptoms indicate the need for somatic follow-up, increased focus on somatic co-morbidities, and planning of long-term care for patients with chronic conditions.

### Strengths and limitations

A strength of the current study is that the data were obtained from a clinical cohort across Norway at two time points with little difference between included participants and those lost to follow-up. The multi-site design and the inclusion of patients from different regions enhances the generalizability of our results to the current health system. By using self-reported measures from a structured interview, we provide information on the experiences of patients with a broad range of somatic symptoms. A limitation is that we did not have independent objective measures of somatic conditions because this was self-reported data, and a one year follow-up may be too short to observe large changes for some conditions. The measure employed in this study to collect data on somatic symptoms is a novel and not specifically validated measure. However, it is worth noting that it has been described and utilized in a previous publication and it drew inspiration from validated somatic symptom questionnaires [16]. Some of the listed symptoms may partly be side effects of the medication itself, such as gastro-intestinal complaints. Although there were no significant differences in the attrition analysis, we cannot rule out the possibility that there may have been differences in other relevant characteristics that were not measured, such as motivational factors. Due to the observational nature of the study, it should be noted that the findings should not be interpreted as establishing a causal relationship between the OMT program and the observed outcomes. Future research should study how specialist healthcare structurally approaches somatic check-ups and responds to the somatic symptoms and conditions reported by patients, including indicators of medication side effects and registry data on somatic health.

## Conclusions

Patients in OMT report a range of somatic conditions and somatic symptoms. Those with a greater somatic symptom burden and greater mental distress at baseline had the largest improvement. Given the wide range of symptoms reported by patients in OMT, including some at high intensity levels, healthcare providers should take into consideration the somatic healthcare needs of individuals in OMT populations.

### Electronic supplementary material

Below is the link to the electronic supplementary material.

Supplementary Material 1

Supplementary Material 2

## Data Availability

The dataset used during the current study is available from the corresponding author on reasonable request.

## Abbreviations

COPD: Chronic obstructive pulmonary disease
CVD: Cardiovascular disease
EuropASI: Addiction Severity Index adapted for European use
HIV: Human immunodeficiency virus
HSCL-25: Hopkins Symptom Checklist 25
IQR: Interquartile range
NorComt: Norwegian Cohort of Patients in Opioid Maintenance Treatment and Other Drug Treatment
OMT: Opioid maintenance treatment
OUD: Opioid use disorder
PHQ-15: Patient Health Questionnaire 15-Item Somatic Symptom Severity Scale
SDS: Severity of Dependence Scale
SUD: Substance use disorder
