# Supplementary material for: Change in self-reported somatic symptoms among patients in opioid maintenance treatment from baseline to 1-year follow-up
Source: BMC Psychiatry. 2024 Feb 21;24:149. doi: 10.1186/s12888-024-05590-w (PMC10882792; doi:10.1186/s12888-024-05590-w)
Supplement: Supplementary file 1 — Supplementary Material 1 [file 12888_2024_5590_MOESM1_ESM.pdf]

Supplementary file 1:
Flowchart describing inclusion of participants and reasons for being lost to follow up at T1

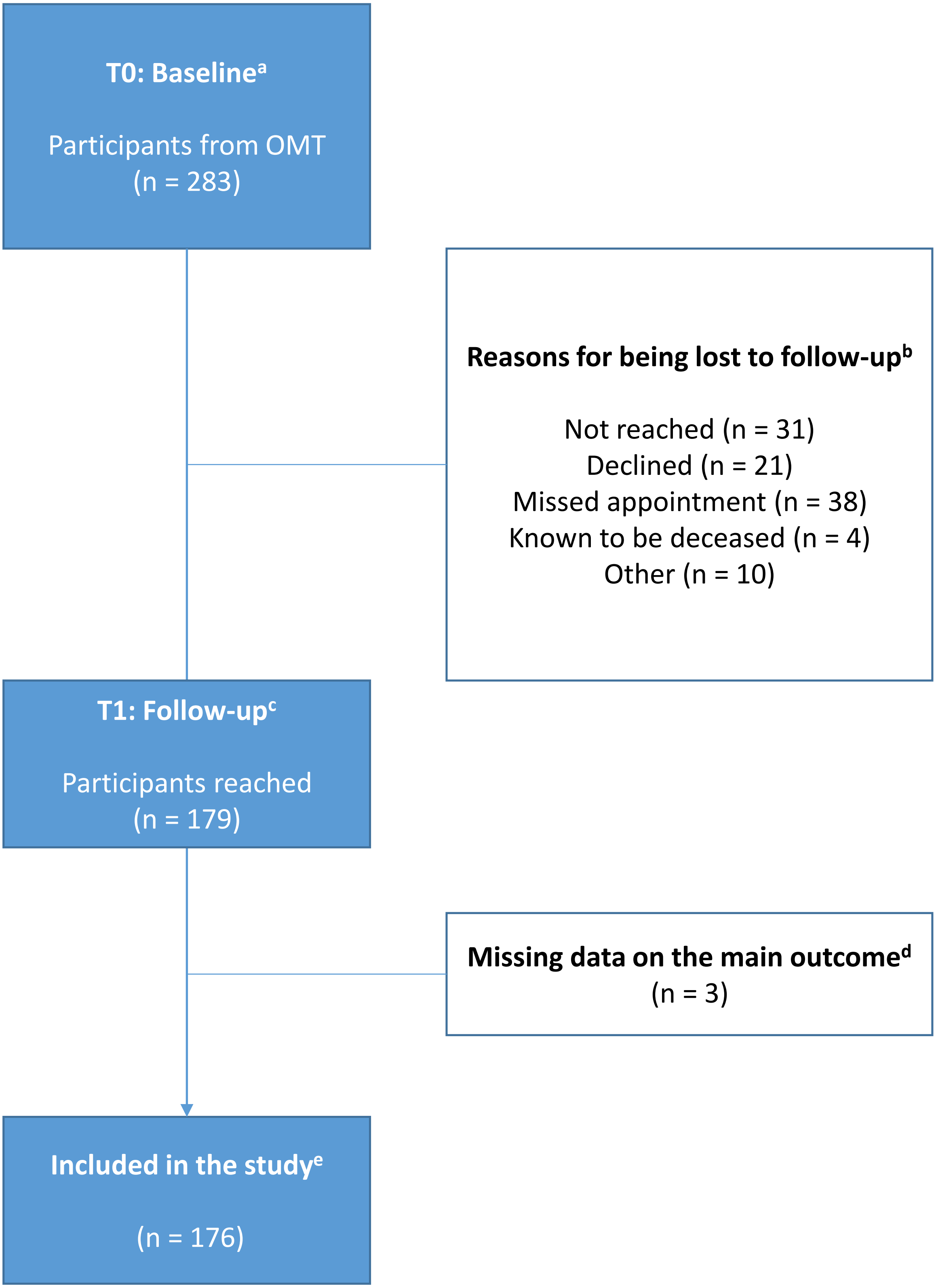

Notes: [a]Number of participants at T0. [b]Reasons for being lost to follow-up for those who participated at T0, but not at T1 (missed appointment includes logistical challenges). [c]Participants reached at follow-up. [d]Number of participants that were excluded due to missing data on the main outcome. [e]Number of participants included in the analysis.
