# Supplementary material for: Change in self-reported somatic symptoms among patients in opioid maintenance treatment from baseline to 1-year follow-up
Source: BMC Psychiatry. 2024 Feb 21;24:149. doi: 10.1186/s12888-024-05590-w (PMC10882792; doi:10.1186/s12888-024-05590-w)
Supplement: Supplementary file 2 — Supplementary Material 2 [file 12888_2024_5590_MOESM2_ESM.pdf]

Supplementary File 2:

Self-reported somatic conditions at T0 among those who were included and those who were lost to follow-up.

Condition type:	Included (N = 176)	Lost to follow-up (N = 107)	p value[a]
	n (%)	n (%)	
Hepatitis C	82 (47)	57 (53)	0.276
Asthma	32 (18)	26 (24)	0.216
High blood pressure	15 (9)	12 (11)	0.439
CVD	13 (7)	7 (7)	0.804
Hepatitis B	12 (7)	9 (8)	0.630
COPD	11 (6)	9 (8)	0.478
Diabetes	5 (3)	1 (1)	0.415
Liver cirrhosis	4 (2)	4 (4)	0.482
HIV	3 (2)	2 (2)	1.000
Cancer	1 (1)	3 (3)	0.151

Notes: CVD, cardiovascular disease; COPD, chronic obstructive pulmonary disease; HIV, human immunodeficiency virus.

Missing data, included group: Hepatitis B, N = 1; Diabetes N = 1; Liver cirrhosis, N = 2. Missing data, lost to follow-up group: Diabetes, N = 1; High blood pressure, N =1; CVD, N = 1; COPD, N = 1; Liver cirrhosis, N = 1; HIV, N = 1; Cancer, N = 1.

[a]p values from Pearson Chi-Square and Fisher's Exact Test. Fisher's Exact test was used when cells in the 2x2 table had an expected count less than 5
